# Sero-epidemiological survey of Crimean-Congo hemorrhagic fever virus in Tunisia

**DOI:** 10.1051/parasite/2016010

**Published:** 2016-03-08

**Authors:** Fares Wasfi, Stuart Dowall, Tayssir Ghabbari, Andrew Bosworth, Mohamed Chakroun, Anitha Varghese, Hanene Tiouiri, Mounir Ben Jemaa, Abir Znazen, Roger Hewson, Elyes Zhioua, Amel Letaief

**Affiliations:** 1 Institut Pasteur de Tunis, Laboratory of Vector Ecology 13 Place Pasteur 1002 Tunis Tunisia; 2 Public Health England, Porton Down Salisbury, Wiltshire SP4 0JG UK; 3 Infectious Diseases Department, Farhat Hached University Hospital 4004 Sousse Tunisia; 4 Infectious Diseases Department, Fatouma Bourguiba University Hospital 5000 Monastir Tunisia; 5 Infectious Diseases Department 3000 Sfax Tunisia; 6 Laboratory of Microbiology 3000 Sfax Tunisia; 7 National Institute of Health Research, Health Protection Research Unit in Emerging and Zoonotic Infections L3 5RF Liverpool UK

**Keywords:** CCHF, CCHF virus, Seroprevalence, Ticks, Tunisia

## Abstract

Crimean-Congo hemorrhagic fever (CCHF) is a tick-borne disease associated with a high case fatality rate and transmitted mainly by *Hyalomma marginatum*. The geographical distribution of *H. marginatum* covers most of the Western Mediterranean basin. We aimed to investigate whether CCHF virus (CCHFv) is circulating in Tunisia. Samples from unexplained acute febrile patients (*n* = 181) and a high risk group of humans, mainly slaughter workers (*n* = 38), were collected in the summer of 2014 and analyzed for exposure to CCHFv using serological tests and real-time RT-PCR. Ticks were collected from Northern and Southern Tunisia during May–June 2014 and examined for the presence of CCHFv by real-time RT-PCR. Of the 181 febrile patients, 5 showed only high titers of IgM suggesting a recent exposure to CCHFv. Among 38 slaughter workers, 2 had IgG anti-CCHFv responses yielding a seroprevalence of 5.2%. No CCHFv was detected in ticks and sera. Our results provide evidence of human exposure to CCHFv in Tunisia.

## Abstract (fr)

La fièvre hémorragique de Crimée-Congo (FHCC) est une maladie transmise par les tiques, associée à un taux de létalité élevé, et transmise principalement par *Hyalomma marginatum*. La répartition géographique de *H. marginatum* couvre la majeure partie du bassin méditerranéen occidental. Nous avons cherché à déterminer si le virus de la FHCC (FHCCv) circule en Tunisie. Les échantillons provenant de patients ayant des fièvres aiguës inexpliquées (*n* = 181) et d’un groupe de personnes à haut risque, principalement des bouchers d’abattoirs (*n* = 38), ont été recueillis pendant l’été 2014 et analysés pour exposition à la FHCC en utilisant un test sérologique et par PCR en temps réel (RT-PCR). Les tiques ont été recueillies du Nord et du Sud de la Tunisie en mai-juin 2014 et examinées pour la présence de FHCCv par RT-PCR. Sur les 181 patients fébriles, 5 ont présenté seulement des titres élevés d’IgM suggérant une exposition récente au FHCCv. Parmi les 38 travailleurs d’abattoirs, 2 avaient des réponses IgG anti FHCCv, indiquant une séroprévalence de 5.2 %. Aucun FHCCv n’a été détecté dans les tiques et les sérums. Nos résultats fournissent des preuves de l’exposition humaine au FHCCv en Tunisie.

## Introduction

Crimean-Congo hemorrhagic fever virus (CCHFv) (family Bunyaviridae, genus *Nairovirus*) is the etiologic agent of Crimean-Congo hemorrhagic fever (CCHF), a severe acute febrile illness characterized by fever and hemorrhage with case fatality rates of 9–50% [6, 28]. CCHF is the most widespread tick-borne disease in the world [12]. It has been reported in more than 30 countries including in Africa [22, 29], Asia [14, 21], the Middle East [25], and has seen a significant increase during the last decade in Eastern Europe [10, 31].

CCHFv is maintained in nature through a transmission cycle involving ticks and vertebrate hosts and is transmitted mainly through the bite of ixodid ticks [7, 9, 12, 26, 30]. CCHFv has been isolated from several ixodid tick species including *Hyalomma marginatum* [10, 26, 30], *Hyalomma dromedarii* [30], *Hyalomma anatolicum* [21, 30], *Hyalomma detritum* [30], *Hyalomma asiaticum* [30], *Rhipicephalus sanguineus* [10], *Rhipicephalus bursa* [26], *Rhipicephalus eversti* [22], *Ixodes ricinus* [10, 26], and *Hyalomma lusitanicum* [7]. In the western Palearctic, *H. marginatum* is considered as the main vector of CCHFv [8]. *H. marginatum* is a two-host tick with larvae and nymphs feeding mainly on small mammals and birds while adults feed on large mammals including livestock [12]. Migratory birds play a significant role in spreading CCHFv between Africa and Europe [15, 17, 24]. While CCHFv is mainly transmitted through tick bites, humans may also become infected through direct contact with the blood or tissues of infected humans or livestock [14, 19, 21, 22]. Thus, farmers and butchers are at high risk for CCHFv infection [21, 22].

CCHFv has previously been isolated from *H. marginatum* collected from migratory birds from Morocco [24]. The geographical distribution of *H. marginatum* also covers Tunisia [20]. Thus, Tunisia could be at risk for CCHFv. In this study, we aimed to perform a sero-epidemiological survey to test for circulation of CCHFv in Tunisia and to assess samples of *Hyalomma* spp. ticks for the presence of viral RNA.

## Materials and methods

### Study sites

**Figure 1.**
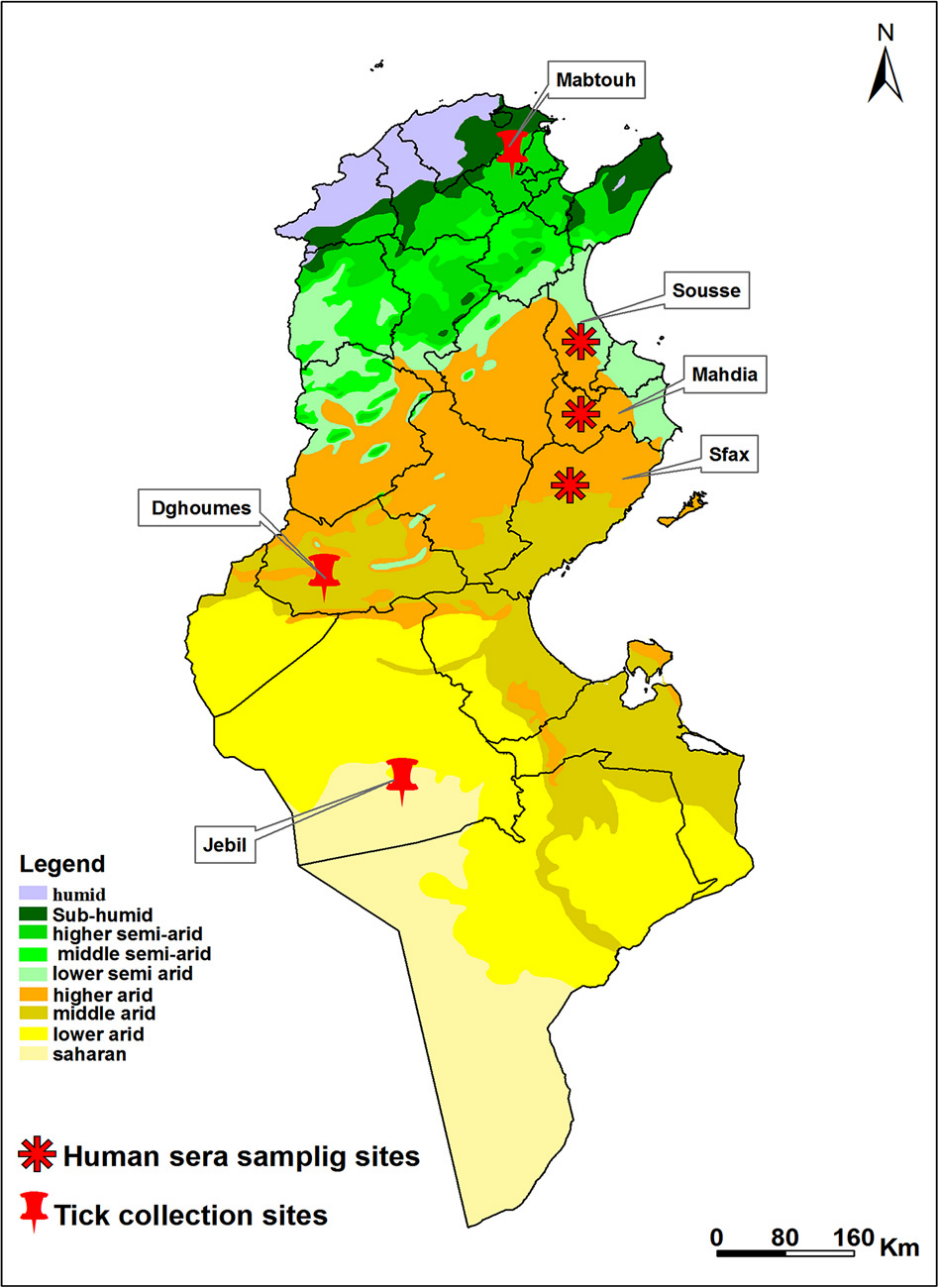
Bioclimatic map of Tunisia showing sites of sera and tick collection

Tunisia covers a wide climatic range, from the Mediterranean climate with its rainy winter in the north to the Saharan climate in the south. The northern part of the country is separated from the south by the Tunisian Ridge. The latter is a range of hills which runs from north-east to south-west for some 220 km, marking the climatic boundary between the Mediterranean north and the dry steppe of Central Tunisia. Between the northern slopes of the Tunisian Ridge and the chains of hills bounding it to the south are extensive plateaus, called the High Tell. The Sahara is separated from the central steppe land by a series of salted areas called chotts (Fig. 1).

### Study population

Samples were collected from patients attending hospitals who had reported fever (*n* = 181). Samples were also actively taken from abattoir workers (*n* = 38) in order to survey evidence of previous exposure among a group designated as high risk. Most of the participants originated from the governorates of Sousse, Sfax, and Mahdia (Fig. 1), and samples were collected during summer 2014. All participants were asked for a history of tick bites and travel history. Samples were collected by vacutainer and the plasma was separated, frozen at −20 °C, and transported to Porton Down, UK for analysis. All the abattoir worker donors were healthy without fever symptoms and the median age was 39 years (range 20–59).

Ticks were collected over the period May–June 2014 from cattle in barns in the region of Mabtouh, a known focus of theileriosis located in North Tunisia (Fig. 1) [11]. In addition, ticks were collected from the National Parks of Dghoumes and Sidi Touil located in Southern Tunisia from the resting sites of oryx (Fig. 1).

### Serological analysis

The collected samples from patients were tested for the presence of IgG and IgM anti-CCHFv by ELISA test according to the instructions of the manufacturer (Vector-Best, Russia). The study was conducted under ethical approval from the Ethics Committee HHS-IRB 00008931, University Hospital Farhat Hached, protocol reference date 08-04-2013.

### Molecular analysis

Ticks and human sera were examined for the presence of CCHFv by real-time RT-PCR [1]. Collected ticks were pooled according to the trapping location with a maximum of 10 specimens per pool for the non-engorged ticks and 2 per pool for engorged ticks, resulting in a total of 46 pools. The pools of ticks were transferred to Precellys tubes containing silica beads and 0.5 mL of Minimum Essential Medium (MEM), homogenized three times at 5600 rpm for 30 s, and finally centrifuged at 6000 rpm/15 min. The total RNA was extracted according to the Qiagen Viral RNA Mini Kit from human sera and tick supernatant. The presence of CCHFv RNA was determined by qualitative real-time reverse transcription PCR performed on the S segment using the combination of the primer pairs CCHFV.S122F (5′CCT TTT TGA ACT CTT CAA ACC 3′)/CCHFV.S1R (5′TCT CAA AGA AAC ACG TGC C 3′) and the CCHFv probe (5′FAM 3′ACT CAA GGK AAC ACT GTG GGC GTA AG-BHQ1), as published previously [1]. The real-time RT-PCR was performed using the SuperScript III Platinum One-Step qualitative RT-PCR system (Invitrogen Life Technologies) according to the manufacturer’s instructions. One high and one low CCHFv viral RNA were used as a positive control. A volume of 5 μL of total RNA extracted was used for CCHFv genome detection.

## Results

Samples collected from patients with acute fever were initially tested according to local protocols for serological reactivity to *Brucella*, *Rickettsia*, and West Nile virus (WNV). All samples in this study were shown to be negative during the testing phase and were then analyzed for CCHF by serological testing. Of a total of 181 sera from febrile patients, 5 (2.7%) showed high titers of IgM anti-CCHFv activity with an absence of IgG (Table 1). None of the patients had traveled to known overseas endemic areas for CCHF nor reported a history of tick bites. The presence of IgG anti-CCHFv was observed in 2 out of 38 slaughter workers, leading to a seroprevalence of 5.2%. No positive cases of CCHFv were detected by RT-PCR analysis.

**Table 1.** List of positive samples, cohorts they belong to, and the associated results.

Sample ID	Type	IgG	IgM	Confirmation	Outcome
TUN/013	Slaughter worker Sera	1:100	Negative	IgG positive	CCHF IgG positive
TUN/024	Slaughter worker Sera	1:100	Negative	IgG positive	CCHF IgG positive
TUN/063	Febrile patient	Negative	1:100	IgM positive	CCHF IgM positive
TUN/080	Febrile patient	Negative	1:100	IgM positive	CCHF IgM positive
TUN/124	Febrile patient	Negative	1:100	IgM positive	CCHF IgM positive
TUN/187	Febrile patient	Negative	1:100	IgM positive	CCHF IgM positive
TUN/190	Febrile patient	Negative	1:100	IgM positive	CCHF IgM positive

A total of 310 ticks were collected over the period May–June 2014 in three different collection sites. Ticks collected from Mabtouh, a known endemic focus for theileriosis, were identified as *Hyalomma scupense*. All ticks collected from Daghounes and Sidi Toui were identified as *H. dromedarii*. No *H. marginatum* were collected. RT-PCR analysis failed to detect the presence of CCHFv RNA, indicating that ticks were not carrying CCHFv.

## Discussion

Five febrile patients tested positive for IgM, which suggests recent infection. Since most patients were discharged, no second blood samples were taken to be tested for the presence of IgG anti-CCHFv. Hemorrhagic manifestations were not observed in any febrile patients. Therefore, as reported in Greece [27] and Turkey [2], it appears that the majority of infections were subclinical.

In our study, a seroprevalence of 5.2% was observed among slaughter workers, suggesting predominance of subclinical forms. Similar seroprevalence concerning high risk populations was reported from other endemic countries: Mauritania (7%) [22]; Greece (4.2%) [27]; Kosovo (4%) [9]; Bulgaria (2.8%) [5]; Turkey (2.3%) [2]; and Iran (12%) [4]. Our results provide more evidence that slaughtering and agricultural activities are significant risk factors for seropositivity [27].

In our study, no CCHFv was detected in the analyzed ticks. *Hyalomma scupense* is the main vector of *Theileria annulata*, etiologic agent of theileriosis in North Africa [11]. *H. scupense* completes its life cycle within the protection of walls and crevices of barns without any other host beside livestock, and subsequently, this tick species has not been implicated in the transmission of CCHFv [23]. *Hyalomma dromedarii* were collected only from the vegetation and from resting sites of oryx and not from livestock (cattle, sheep, or dromedary), known to be the main amplifying hosts for CCHFv [12]. *Hyalomma marginatum*, a main vector of CCHFv, is located mainly in the Northwestern part of Tunisia [20], an area that was not investigated during the study period. Thus, further studies concerning *H. marginatum* are needed.

Our results suggest circulation of CCHFv in Tunisia. The seroprevalence observed among the high risk population was not unexpected mainly because of the circulation of CCHFv among populations of *H. marginatum* in North Africa. To date, no clinical cases of CCHF (fever followed by hemorrhagic manifestations) have been observed in North Africa. It is of major epidemiological importance to point out that the detection of CCHFv in *H. marginatum* collected from birds in Morocco and in *H. aegyptium* collected from tortoises in Algeria provides strong evidence that the virus is transmitted in North Africa mainly by ticks belonging to the genus *Hyalomma* [13, 24]. The detection of the non-pathogenic CCHFv strain AP92 (Europe 2) in *H. aegyptium* collected from tortoises in Algeria [13] could explain in part the absence of clinical cases in the Western Mediterranean basin [27]. This hypothesis is corroborated by the fact that the low-pathogenic viruses found in Greece are phylogenetically close to known sequences from African strains [17]. The geographical distribution of clinical cases of CCHF is limited to the Western Balkans. However, the geographical distribution of *H. marginatum* covers significantly larger areas including Spain, Southern Italy, and North Africa. The absence of human clinical cases in Southern Europe could be explained by the lack of movements of livestock coupled with the apparent lack of movements of wild hosts for ticks between the Western Mediterranean basin and the Balkans, leading to a lack of connectivity between eastern and western populations of the vector and virus [8]. However, it is of major epidemiological importance to point out that during the Eid-al-Adha festival, every year Tunisia imports up to 100,000 sheep from several countries including Romania where 27.8% had IgG antibodies specific to CCHFv [3]. During these festivals, the vast majority of Tunisians, as most Muslims, sacrifice sheep by themselves with guidelines focusing only on echinococcosis but not on CCHF prevention. In addition, the dates of the Islamic Calendar for Eid-al-Adha are drifting 10 days each year according to the Gregorian Calendar. In the next 10–15 years, Eid-al-Adha will occur in summer-autumn when CCHFv is more prevalent. Therefore, movement of livestock and wild hosts for ticks (mainly migratory birds) between North Africa and the Balkans, coupled with slaughtering activities during the Eid-al-Adha festival, particularly during summer-autumn, may lead to an increased risk of CCHFv in Tunisia [16]. Thus, preparedness for such a risk is of high importance [18].

In conclusion, our results provide strong evidence of circulation of CCHFv in Tunisia for the first time. Thus, further studies are recommended to be carried out on livestock, high risk groups of humans, birds, and ticks for a better understanding of the dynamic transmission of CCHFv in Tunisia and surrounding countries in North Africa.

## Conflict of interest

The authors declare no conflict of interest in relation with this paper.
